# Delay of cognitive gamma responses in Alzheimer's disease

**DOI:** 10.1016/j.nicl.2016.01.015

**Published:** 2016-01-20

**Authors:** Erol Başar, Derya Durusu Emek-Savaş, Bahar Güntekin, Görsev G. Yener

**Affiliations:** a Brain Dynamics, Cognition and Complex Systems Research Center, Istanbul Kultur University, Istanbul 34156, Turkey; b Department of Psychology, Dokuz Eylül University, Izmir 35160, Turkey; c Department of Neurosciences, Dokuz Eylül University, Izmir 35340, Turkey; d Brain Dynamics Multidisciplinary Research Center, Dokuz Eylül University, Izmir 35340, Turkey; e Department of Neurology, Dokuz Eylül University Medical School, Izmir 35340, Turkey

**Keywords:** Event-related, Sensory-evoked, Oscillation, Gamma, Alzheimer's disease, EEG, P300

## Abstract

Event-related oscillations (EROs) reflect cognitive brain dynamics, while sensory-evoked oscillations (SEOs) reflect sensory activities. Previous reports from our lab have shown that those with Alzheimer's disease (AD) or mild cognitive impairment (MCI) have decreased activity and/or coherence in delta, theta, alpha and beta cognitive responses. In the current study, we investigated gamma responses in visual SEO and ERO in 15 patients with AD and in 15 age-, gender- and education-matched healthy controls. The following parameters were analyzed over the parietal-occipital regions in both groups: (i) latency of the maximum gamma response over a 0–800 ms time window; (ii) the maximum peak-to-peak amplitudes for each participant's averaged SEO and ERO gamma responses in 3 frequency ranges (25–30, 30–35, 40–48 Hz); and (iii) the maximum peak-to-peak amplitudes for each participant's averaged SEO and ERO gamma responses over a 0–800 ms time block containing four divided time windows (0–200, 200–400, 400–600, and 600–800 ms). There were main group effects in terms of both latency and peak-to-peak amplitudes of gamma ERO. However, peak-to-peak gamma ERO amplitude differences became noticeable only when the time block was divided into four time windows. SEO amplitudes in the 25–30 Hz frequency range of the 0–200 ms time window over the left hemisphere were greater in the healthy controls than in those with AD. Gamma target ERO latency was delayed up to 138 ms in AD patients when compared to healthy controls. This finding may be an effect of lagged neural signaling in cognitive circuits, which is reflected by the delayed gamma responses in those with AD. Based on the results of this study, we propose that gamma responses should be examined in a more detailed fashion using multiple frequency and time windows.

## Highlights

• Gamma responses elicited by sensory or cognitive stimulation were investigated.
• Three frequency bands and four time windows were analyzed.
• Alzheimer's disease (AD) patients and healthy controls were included in the study.
• AD patients showed delayed cognitive gamma responses.
• This delay was possibly due to lagged neural signals in cognitive circuits.

## 1 Introduction

Alzheimer's disease (AD) is the most common dementing illness. In the majority of cases, mild cognitive impairment (MCI) is considered to be prodromal AD (Petersen et al., 2001, Rasquin et al., 2005, Alexopoulos et al., 2006). Current diagnostic methods are heavily weighted by the amyloid and tau levels in the cerebrospinal fluid (CSF) and by volumetric magnetic resonance imaging (MRI) measurements. The full potential of electrophysiological methods for use in predicting (Cichocki et al., 2005, Babiloni et al., 2006a, Rossini et al., 2006), diagnosing (Yener et al., 1996, Polich and Herbst, 2000, Jeong, 2004, Babiloni et al., 2006b, Karrasch et al., 2006), and monitoring treatment or progress (Jelic et al., 2000, Dauwels et al., 2010) in AD/MCI patients has not been fully examined in routine clinical practice.

Brain oscillatory responses can be used for the non-invasive analysis of local neuronal synchronization, cortico-cortical connectivity, and coherence of oscillations (Rossini et al., 2007). Cognitive stimuli can elicit event-related oscillations (EROs), which is a powerful technique with high temporal resolution. ERO has been described as a useful tool for detecting subtle abnormalities of cognitive processes (Basar, 1980, Başar, 2004).

In our previous work, we explored ERO, sensory-evoked oscillations (SEOs), and the evoked or event-related coherence of AD/MCI patients using visual and auditory sensory modalities (Yener et al., 2008, Yener et al., 2009, Yener et al., 2012, Güntekin et al., 2008, Basar et al., 2010, Yener and Başar, 2010). The term “event-related” is used for a “potential” that is elicited after a cognitive task, while the term “sensory-evoked” is used for a “potential” that is elicited after a sensory stimulus (Başar et al., 1997).

The history of gamma activity began in the 1940s (Adrian, 1942). In subsequent years, Freeman (1975) and Başar et al., 1975a, Başar et al., 1975b, Başar et al., 1975c indicated that gamma oscillatory responses reflect a wide variety of functions. In 1972, Başar and Özesmi introduced the terminology “gamma response” to describe hippocampal gamma band activity elicited by external stimuli in cats. In human studies, Galambos (1981) later indicated that there are sensory and cognitive correlates of gamma responses. Gamma oscillatory responses are selectively distributed in the brain, but they do not appear to reflect a specific function in the nervous system. Gamma activity has been related to both sensory and cognitive responses from the cortex, hippocampus, thalamus, and reticular formations in both animal and human brains (Başar, 2013). Thus, it can be hypothesized that gamma-band synchronization is most likely a fundamental process in all brain functions [see Başar et al., 1999, Başar et al., 2013 and Başar-Eroglu et al. (1996a)].

In the last decade, there have been several studies published on gamma activity in cognitive impairment, especially in schizophrenia. Most of the results related to these functions indicate a decrease in gamma responses. It is important to note that studies on healthy participants and participants with cognitive impairment have contradictory results and interpretations. Gamma oscillatory responses have been found to play role in perception, attention and memory processes, object recognition, face recognition and emotional paradigms (Güntekin and Başar, 2014, Keil et al., 1999, Busch et al., 2004, Busch et al., 2006, Tallon-Baudry et al., 1998, Gruber et al., 2004, Herrmann et al., 2004a, Müller and Keil, 2004, Senkowski and Herrmann, 2002; for further information on gamma responses please see reviews Başar, 2013, Başar-Eroglu et al., 1996b, Herrmann et al., 2004b, Jensen et al., 2007, Singer, 1999, Tallon-Baudry and Bertrand, 1999). In the literature, gamma responses have mostly been analyzed in single frequency and single time windows. There are few studies analyzing gamma responses in multiple frequency and time windows. However, our recent study (Başar et al., 2015) showed that analyzing the gamma responses in multiple frequency and time windows is extremely important. Başar et al. (2015) showed that, especially during cognitive paradigm, there are at least 3–4 phase/time-locked gamma responses in the 25–45 Hz frequency windows that occur in multiple time windows (between 0 and 800 ms). In most cases cognitive responses are late (200–400 ms, 400–600 ms), and they depict higher frequencies. Since there were many differences in the gamma responses in multiple frequency and time domains, this manuscript aims to analyze gamma responses in multiple time and frequency windows.

The literature regarding gamma responses in AD or MCI indicates that auditory steady state gamma responses with amplitudes of 40 Hz are increased in AD (Osipova et al., 2006) and MCI (van Deursen et al., 2011) patients when compared to controls. Another study comparing gamma activity during the N-back paradigm in stable and progressive MCI patients indicates that the progressive MCI group has lower average changes in gamma values (Missonnier et al., 2010). In the present study, we aimed to analyze gamma responses elicited by sensory or cognitive stimulation in AD patients using multiple frequency bands and many time windows. A new strategy was used that involved the analysis of three gamma frequency bands within four time windows. We hypothesized that cognitive gamma responses would be delayed in AD due to lagged neural signals in cognitive circuits.

## 2 Materials and methods

### 2.1 Participants

A total of 15 probable mild AD patients who were diagnosed according to DSM-IV and NINCDS-ADRDA criteria and 15 age-, gender-, and education-matched healthy controls were consented to participate in the study. All AD patients were within the first year of their diagnosis, and six of these patients were taking a cholinesterase inhibitor (donepezil, rivastigmine). The mean age of the healthy controls was 67.47 years (SD 4.14), while the mean age of the AD patients was 67.53 years (SD 6.48). The mean educational years was 8.73 (SD 6.03) for the healthy controls and 8.67 (SD 4.75) for the AD patients. There were 7 females and 8 males in each group. The mini-mental state examination (MMSE) scores ranged between 28 and 30 for the healthy controls and 16–27 for the AD patients, out of a possible 30 points. The general demographic and clinical features of both groups are shown in Table 1. All participants and/or their relatives provided informed consent for the study, which was approved by the local ethical committee.

### 2.2 Acquisition of visual sensory-evoked oscillations (SEOs) and visual event-related oscillations (EROs)

#### 2.2.1 Sensory-evoked oscillations (SEOs)

A visual sensory paradigm was administered to each participant. A white screen with 40 cd/cm^2^ luminance was used as the stimulus. The duration of the stimulation was 1000 ms. Sixty stimulation signals were applied, and the inter-stimulus intervals varied randomly between 3 and 7 s.

#### 2.2.2 Event-related oscillations (EROs)

A classical visual oddball paradigm was administered to all participants. There were 40 target and 80 standard stimulations. The probability of the target stimuli was 0.33. A white screen with a 10 cd/cm^2^ luminance was used for standard signal stimulation and 40 cd/cm^2^ was used for the target signals. The duration of the stimulation was 1000 ms. The light appeared at full size on a 22-inch computer monitor with a refresh rate of 75 Hz. The target stimuli were embedded randomly within a series of standard stimuli in all of the paradigms. The task required the target stimuli to be counted, and the inter-stimulus interval varied randomly between 3 and 7 s.

Ten of the healthy controls counted 40 target stimulations; three of the healthy controls made one mistake while counting the target stimulation; and two made more than one mistake. Eight of the AD patients counted 40 target stimulations; two of the AD patients made one mistake; and five of them made more than one mistake. There was no significant difference between groups in terms of counting the target stimulation (p = 0.389).

### 2.3 Electrophysiological recording

EEGs were recorded according to the International 10–20 system using 30 Ag-AgCl electrodes mounted in an elastic cap (Easy-cap). Two additional linked Ag-AgCl earlobe electrodes (A1 + A2) were used as references. The electrooculogram (EOG) was registered from both the medial upper and the lateral orbital rim of the right eye. All electrode impedances were less than 10 kΩ. The EEG was amplified with a BrainAmp 32-channel DC system with band limits of 0.01–250 Hz, and a sampling rate of 500 Hz was used.

Prior to averaging the data, epochs containing artifacts were rejected by a manual off-line technique (i.e., single sweep EOG recordings were visually studied, and trials with eye movement or blink artifacts were rejected). Sweep numbers were randomly equalized between the target and simple visual stimulation.

### 2.4 Measurements

Gamma SEO and ERO responses were digitally filtered in three gamma ranges measured from P_3_, P_z_, P_4_, O_1_, O_z_ and O_2_ locations and using filter limits of 25–30, 30–35, and 40–48 Hz. The slope of the band-pass filter was 48 dB/octave. Parietal and occipital locations were chosen as the regions of interest since gamma activity has been reported to produce significant results upon visual stimulation, especially in the posterior parts of the brain (Başar et al., 2015, Busch et al., 2006, Castelhano et al., 2013, Sakowitz et al., 2001). In our previous study (Başar et al., 2015), we investigated multiple gamma oscillatory responses in healthy young subjects upon presentation of simple visual stimuli and visual oddball paradigm. Gamma responses in multiple time and frequency windows were analyzed from the frontal, central, parietal and occipital electrodes. This manuscript showed that the most significant results were found in the parietal and occipital regions. Accordingly, in the present manuscript, parietal and occipital locations were chosen as the regions of interest.

First, the peak-to-peak amplitude and the latency of the maximum gamma oscillatory activity over a 0–800 ms time window were measured in 3 gamma ranges (25–30, 30–35, 40–48 Hz), which were chosen from the power spectra evaluation. Then, the peak-to-peak amplitude values of the gamma responses were measured in 3 frequency ranges (25–30 Hz, 30–35 Hz, 40–48 Hz) over 4 time windows (0–200 ms, 200–400 ms, 400–600 ms, 600–800 ms). These time periods were chosen based on the evaluation of the grand average pictures. In our previous study, we analyzed the multiple gamma responses of healthy young subjects upon presentation of visual sensory stimulation and visual oddball paradigm (Başar et al., 2015). In that study, we showed that there were multiple gamma responses in different time domains. Late gamma responses mostly appeared during cognitive load. The difference between cognitive stimulation and sensory stimulation in the parietal locations was seen mostly in the 600–800 ms time window. Furthermore, we showed that 40–48 Hz gamma response oscillations were significantly greater in the third time window (400–600 ms) upon application of target stimulation in comparison to simple light stimulation. The present study therefore takes our previous findings into consideration. The observations of subject averages as well as the statistical results of the present study show that AD patients have significantly later responses than healthy controls. It would not be possible to see this difference without analyzing the gamma responses in different time windows, including the late responses (400–600 ms, 600–800 ms).

Three different measurements were performed in order to determine the entire dynamic properties of the gamma responses. An earlier report from our lab (Başar et al., 2015) showed that there are multiple gamma responses over different time and frequency windows during cognitive stimulation. Therefore, it is more appropriate to analyze gamma responses over multiple time and frequency domains. In addition, we (Başar et al., 2015) previously showed that cognitive stimulation elicited more gamma responses over different time and frequency windows than did simple sensory stimulation.

### 2.5 Statistical analysis

Statistical analyses were performed with Statistica Software. Repeated measures ANOVA was used for statistical analysis. Repeated measures ANOVAs were run separately for three different measurements as follows: (1) maximum peak-to-peak gamma amplitudes for three different gamma frequency ranges (25–30 Hz, 30–35 Hz, 40–48 Hz) over a 0–800 ms time window; (2) maximum peak-to-peak gamma amplitudes for three different frequency ranges (25–30 Hz, 30–35 Hz, 40–48 Hz) and over four different time windows (0–200 ms, 200–400 ms, 400–600 ms, 600–800 ms); and (3) latency of maximum gamma amplitude for three different gamma frequency ranges (25–30 Hz, 30–35 Hz, 40–48 Hz) over a 0–800 ms time window.

The repeated measures ANOVA analysis of “maximum peak-to-peak gamma amplitudes for three different gamma frequency ranges over a 0–800 ms time window” included 2-level GROUP (AD patients and healthy controls) as between-subject factors, and frequency range (FR [3 levels] = 25–30 Hz, 30–35, 40–48 Hz), anterior–posterior distribution (AP [2 levels] = parietal, occipital), and lateral distribution (LAT [3 levels] = left, midline, right) as within-subject factors.

The repeated measures ANOVA analysis for “maximum peak-to-peak gamma amplitudes for three different frequency ranges and over four different time windows” included 2-level GROUP (AD patients and healthy controls) as between-subject factors, and time window (TW [4 levels] = 0–200 ms, 200–400 ms, 400–600 ms, 600–800 ms), frequency range (FR [3 levels] = 25–30 Hz, 30–35, 40–48 Hz), anterior–posterior distribution (AP [2 levels] = parietal, occipital), and lateral distribution (LAT [3 levels] = left, midline, right) as within-subject factors.

The repeated measures ANOVA analysis for “latency of maximum gamma amplitude for three different gamma frequency ranges over a 0–800 ms time window” included 2-level GROUP (AD patients and healthy controls) as between-subject factors, and frequency range (FR [3 levels] = 25–30 Hz, 30–35, 40–48 Hz), anterior–posterior distribution (AP [2 levels] = parietal, occipital), and lateral distribution (LAT [3 levels] = left, midline, right) as within-subject factors.

These analyses were carried out for both SEO and ERO responses separately. In summary, six different repeated measures ANOVAs were run as described above and listed below:

1) SEO: “maximum peak-to-peak gamma amplitudes for three different gamma frequency ranges (25–30 Hz, 30–35 Hz, 40–48 Hz) over a 0–800 ms time window”
2) SEO: “maximum peak-to-peak gamma amplitudes for three different frequency ranges (25–30 Hz, 30–35 Hz, 40–48 Hz) and over four different time windows (0–200 ms, 200–400 ms, 400–600 ms, 600–800 ms)”
3) SEO: “latency of maximum gamma amplitude for three different gamma frequency ranges (25–30, 30–35, 40–48 Hz) over a 0–800 ms time window”
4) ERO in response to target stimulation: “maximum peak-to-peak gamma amplitudes for three different gamma frequency ranges (25–30 Hz, 30–35 Hz, 40–48 Hz) over a 0–800 ms time window”
5) ERO in response to target stimulation: “maximum peak-to-peak gamma amplitudes for three different frequency ranges (25–30 Hz, 30–35 Hz, 40–48 Hz) and over four different time windows (0–200 ms, 200–400 ms, 400–600 ms, 600–800 ms)”
6) ERO in response to target stimulation: “latency of maximum gamma amplitude for three different gamma frequency ranges (25–30, 30–35, 40–48 Hz) over a 0–800 ms time window”

Greenhouse–Geisser corrected p-values are reported. Post-hoc comparisons were analyzed with t-tests used with Bonferroni correction. Levels of p < 0.05 were considered significant for all comparisons.

## 3 Results

### 3.1 Visual sensory-evoked oscillation (SEO) amplitudes

#### 3.1.1 Maximum peak-to-peak gamma SEO amplitudes at three frequency ranges over a 0–800 ms time window

There was no main GROUP effect on visual SEO amplitudes for the three frequency ranges over a 0–800 ms time window. However, there was a main FR effect [F_2.56_ = 14.112; p = 0.000], with lower amplitudes in the 40–48 Hz frequency range than in both the 25–30 Hz (p = 0.000) and the 30–35 Hz (p = 0.024) frequency ranges. It is also important to keep in mind that higher frequency brain oscillations almost always show lower amplitudes.

#### 3.1.2 Maximum peak-to-peak gamma SEO amplitudes at three frequency ranges over four time windows

Repeated measures ANOVA revealed main effects for FR [F_2.56_ = 17.244; p = 0.000], TW [F_3.84_ = 10.454; p = 0.000] and AP [F_1.28_ = 14.203; p = 0.001]. Interaction-effects for FR × TW [F_6.168_ = 2.956; p = 0.037], FR × AP [F_2.56_ = 13.685; p = 0.000], and FR × TW × LAT × GROUP [F_12.336_ = 2.503; p = 0.03] were also observed.

Post-hoc analysis of FR revealed that the 25–30 Hz frequency range had a significantly higher amplitude than the 30–35 Hz (p = 0.000) and the 40–48 Hz (p = 0.001) frequency ranges. Moreover, the 30–35 Hz frequency range had a significantly higher amplitude than the 40–48 Hz (p = 0.01).

Post-hoc analysis for TW revealed that the 0–200 ms time window had the highest amplitude when compared to other three time windows (p = 0.001, p = 0.003, p = 0.045, respectively). The 600–800 ms time window had the second highest amplitude, while both the 200–400 ms and the 400–600 ms time windows had the lowest amplitudes.

Post-hoc analysis for AP indicated that the parietal locations had significantly higher gamma SEO amplitudes than the occipital electrodes (p = 0.001).

Post-hoc analysis of FR × AP revealed that the parietal 25–30 Hz gamma SEO amplitude was significantly higher than all of the others (p < 0.001 for all comparisons), while 40–48 Hz gamma SEO amplitude in both the parietal and the occipital leads was the lowest.

**Fig. 1:**
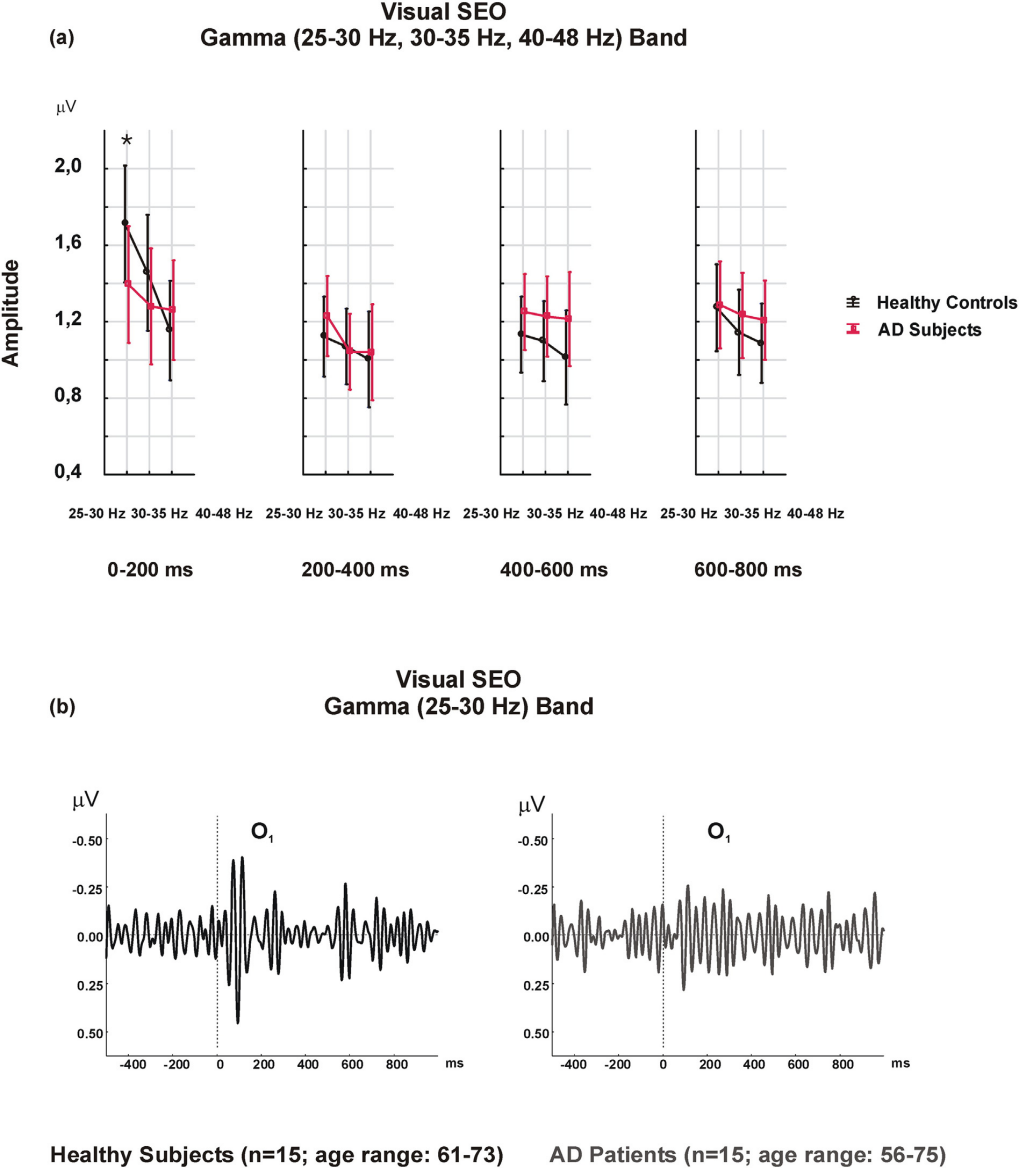
a) Visual gamma SEO responses reveal higher amplitude values in healthy controls with a FR × TW × LAT × GROUP interaction-effect over the left hemisphere, in a 25–30 Hz frequency range, and over a 0–200 ms time window (*p < 0.05). b) Grand averages of visual gamma SEO in the 25–30 Hz frequency range in the left occipital location indicate higher amplitudes over the 0–200 ms time window in healthy controls.

The post-hoc analysis of FR × TW × LAT × GROUP indicated non-significant results. However, the differences between the healthy controls and AD patients were greatest in the 0–200 ms time window. The healthy controls had higher gamma amplitudes than AD patients, especially in the 25–30 Hz frequency range, over the 0–200 ms time window, and in the left hemisphere (mean value = 1.71 μV; SD = 0.72 μV for healthy controls) (mean value = 1.39 μV, SD = 0.57 μV for AD patients) (Fig. 1).

### 3.2 Gamma target ERO amplitudes

#### 3.2.1 Maximum peak-to-peak gamma target ERO amplitudes in three frequency ranges over a 0–800 ms time window

There was no main GROUP effect on maximum peak-to-peak gamma target ERO amplitudes in three frequency ranges over a 0–800 ms time window. However, there was a main FR effect [F_2.56_ = 14.569; p = 0.000], with higher amplitudes in the 25–30 Hz frequency range than in the 30–35 Hz (p = 0.003) and the 40–48 Hz (p = 0.001) frequency ranges. Moreover, the 30–35 Hz had higher amplitudes than did the 40–48 Hz (p = 0.009).

**Fig. 2:**
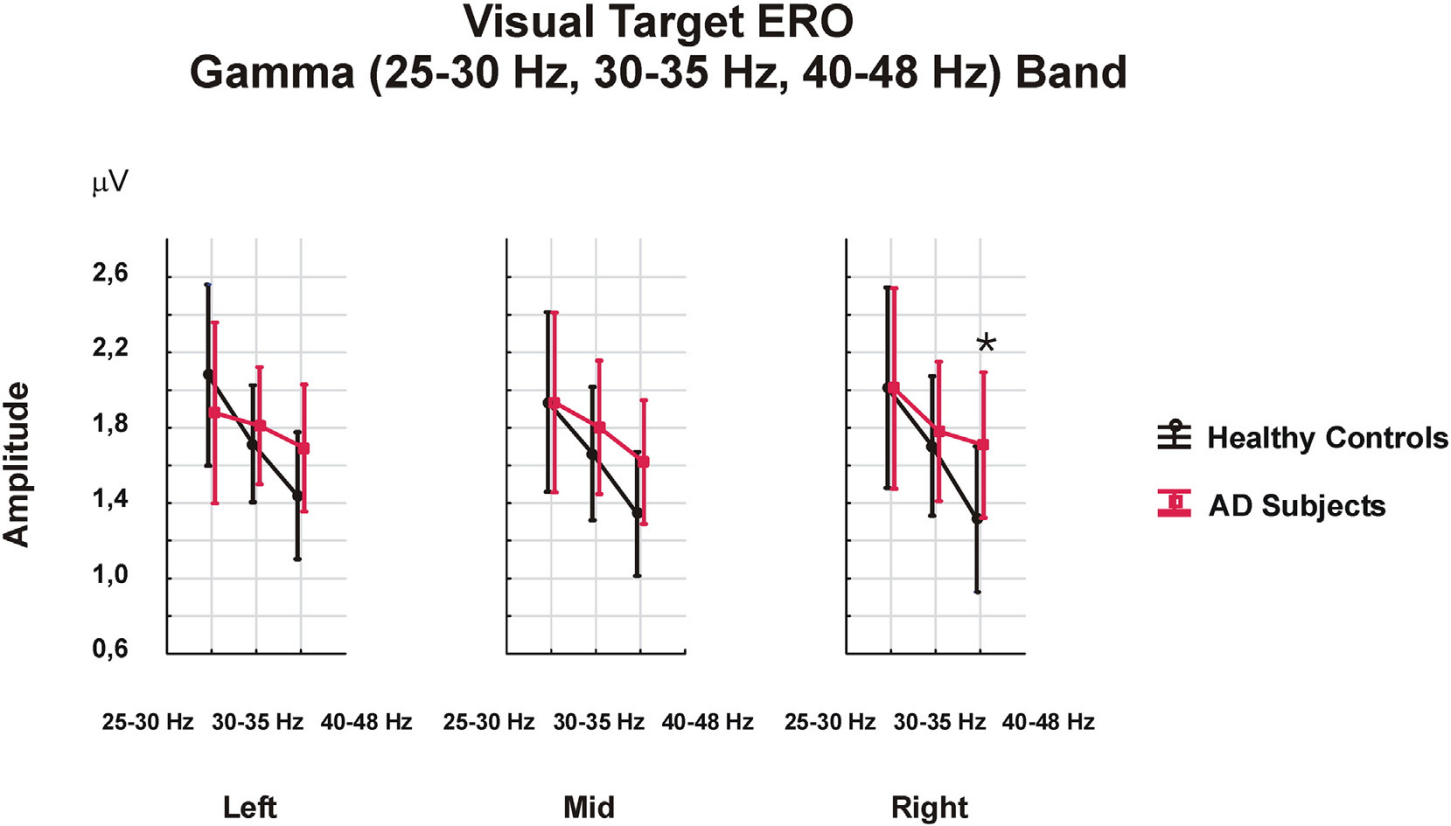
Visual gamma target ERO responses over a 0–800 ms time window show a significant FR × LAT × GROUP interaction-effect, indicating higher amplitude values in AD patients in the right hemisphere in the 40–48 Hz frequency range (*p < 0.05).

A significant interaction-effect for the FR × LAT × GROUP [F_4.112_ = 3.379; p = 0.022] was also observed, and post-hoc analysis showed that the difference between healthy controls and AD patients was greatest in the 40–48 Hz frequency range over the right hemisphere (p = 0.04). AD patients had a mean value of 1.708 μV (SD 0.646 μV), while the healthy controls had a mean value of 1.314 μV (SD 0.408 μV) (Fig. 2).

#### 3.2.2 Maximum peak-to-peak gamma target ERO amplitudes in three frequency ranges over four time windows

There was a main GROUP effect in maximum peak-to-peak gamma target ERO amplitudes in three frequency ranges over four time windows [F_1.28_ = 4.259; p = 0.048], with higher values in AD compared to healthy controls [mean 1.423 μV (SD 0.67) and 1.145 μV (SD 0.54), respectively].

A main FR effect [F_2.56_ = 8.790; p = 0.004] was also observed, with higher amplitudes in the 25–30 Hz frequency range than in both the 30–35 Hz and 40–48 Hz frequency ranges (p = 0.006, p = 0.013, respectively). The 30–35 Hz and 40–48 Hz frequency ranges did not differ. In addition, there was a main TW effect [F_3.84_ = 11.146; p = 0.000], with the 0–200 ms time window showing higher amplitudes than the 200–400 ms, the 400–600 ms, and the 600–800 ms time windows (p = 0.000, p = 0.001, p = 0.006, respectively). The data from the three later time blocks did not differ.

There was an interaction-effect for TW × GROUP [F_3.84_ = 4.744; p = 0.009]. Post-hoc comparisons indicated that for healthy controls, the 0–200 ms time window elicited a higher gamma response than each of the other time windows, namely the 200–400 ms (p = 0.0003), the 400–600 ms (p = 0.000007), and the 600–800 ms (p = 0.00002) windows. However, no significant differences between time windows were observed in AD patients.

An interaction-effect for FR × TW [F_6.168_ = 6.921; p = 0.000] was also observed. Post-hoc comparisons indicated that the 25–30 Hz frequency range in the 0–200 ms time window was higher than all of the other time window × frequency range combinations (p < 0.001 for all comparisons). Furthermore, the 30–35 Hz frequency range in the 0–200 ms window was the second highest time window × frequency range combination, and was higher than any of the other combinations.

### 3.3 Visual gamma SEO latency in three frequency ranges over a 0–800 ms time window

There was no main GROUP effect on latency of maximum gamma SEO responses in three frequency ranges over a 0–800 ms time window. The mean latency of the maximum gamma response in the overall frequency ranges was 312.79 ms (SD 235.29 ms) in healthy controls and 363.61 ms (SD 251.49 ms) in AD patients.

There was an interaction-effect on AP × LAT [F_2.56_ = 3.411; p = 0.042]. Post-hoc comparisons revealed that the right parietal location had earlier gamma responses [317.98 (SD 228.16) ms] than the left parietal location [385.96 (SD 249.17) ms] (p = 0.037).

### 3.4 Visual gamma target ERO latency at three frequency ranges over a 0–800 ms time window

**Fig. 3:**
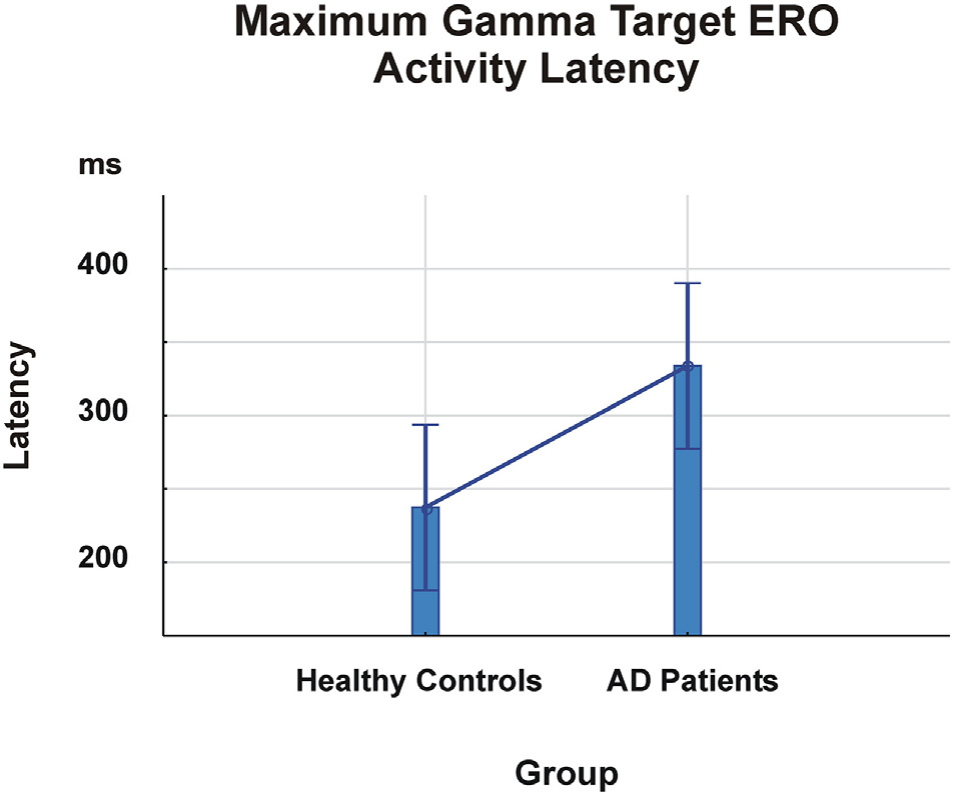
The mean latency values of the maximum gamma ERO in the overall gamma frequency ranges in healthy controls and AD patients indicate that AD patients display later gamma responses. The mean values include the latency values of all electrodes.

There was a main GROUP effect on latency of maximum gamma ERO responses in 3 frequency ranges over the 0–800 ms time window [F_1.28_ = 6.132; p = 0.02]. The mean value of latency for the maximum gamma response in the overall frequency ranges was 237.38 ms (SD 212.04 ms) in the healthy controls and 333.87 ms (SD 241.12 ms) in the AD patients, which was significantly different (p < 0.05). As shown in Fig. 3, there are significant delays in the cognitive gamma responses of the AD patients.

Moreover, there was a main FR effect [F_2.56_ = 3.645; p = 0.038], with earlier gamma latency responses in the 25–30 Hz frequency range than in the 40–48 Hz frequency range (p = 0.027).

Table 2 presents the ANOVA results of the maximum peak-to-peak amplitudes and latency values of event-related and evoked gamma oscillations.

Across all frequency ranges, the mean value of latency of gamma ERO in healthy controls appeared 39–138 ms before those in the AD group.

**Fig. 4:**
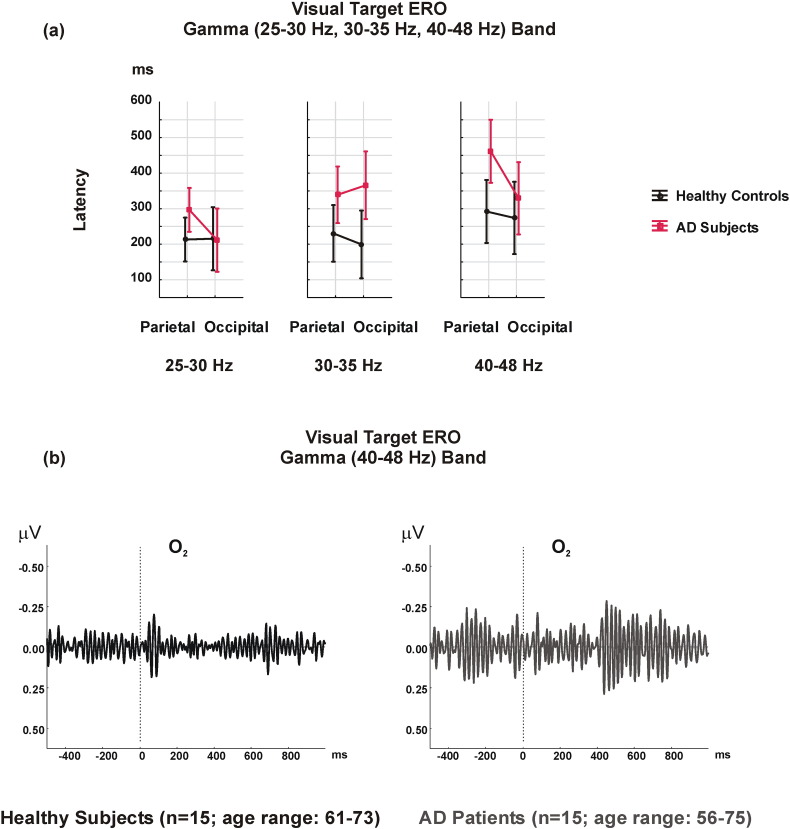
a) The latency of the maximum gamma target ERO responses over parietal and occipital electrodes in the 0–800 ms time domain for the 3 frequency ranges shows a main GROUP effect, with later gamma responses in AD patients compared to healthy controls. b) Grand averages of the visual gamma ERO responses in the 40–48 Hz frequency range over the right occipital location indicate delayed visual gamma ERO responses in the AD group.

The mean latency values of maximum cognitive gamma responses in parietal and occipital locations for 25–30 Hz were 213.1 (SD 187.6) ms and 215.4 (SD 207.3) ms in healthy controls and 296.6 (SD 208.7) ms and 211.3 (SD 197.2) ms in AD patients. These values were 230.4 (SD 194.95) ms and 199.4 (SD 178.98) ms in healthy controls and 339.02 (SD 236.3) ms and 365.8 (SD 256.7) ms in AD patients in the 30–35 Hz frequency range; and 292.1 (SD 248.8) ms and 273.9 (SD 240.2) ms in healthy controls and 461.4 (SD 242.7) ms and 329.1 (SD 240.5) ms in AD patients in the 40–48 Hz frequency range (Fig. 4).

Fig. 4 suggests that there was a delay in the cognitive gamma responses of AD patients within the divided frequency ranges. The gamma ERO responses of the AD group were delayed 84 ms in parietal locations in the 25–30 Hz frequency range, 108 ms in parietal and 166 ms in occipital locations in the 30–35 Hz frequency range, and 169 ms in parietal and 55 ms in occipital locations in the 40–48 Hz frequency range when compared to healthy controls (Fig. 4a).

## 4 Discussion

In this paper, we investigated both sensory and cognitive gamma responses in three frequency ranges over four time windows. Our results indicated significant and diverse differences between AD patients and healthy controls, implying a separation of sensory and cognitive gamma response-related circuits in AD. Our earlier work on the delta band range in AD (Yener and Başar, 2010) and MCI (Yener et al., 2014) also demonstrated the involvement and separation of sensory and cognitive circuits in AD/MCI.

Earlier results from our laboratory showed differences in gamma responses from healthy controls in three frequency bands and over four time windows; these data imply that diverse sensory/cognitive circuits exist (Başar et al., 2015). Cognitive impairment can differentially alter these circuits.

### 4.1 Sensory gamma responses

Sensory gamma responses yielded an interaction-effect on frequency ranges × time window × laterality × group, indicating greater amplitudes in healthy controls than in AD patients. Fig. 1b might provoke the idea that AD patients produced larger temporal inter-individuality across time windows compared to more burst-like temporal signal in healthy controls. This sustained character of sensory gamma responses in AD patients may potentially differentiate these two groups.

### 4.2 Cognitive gamma responses

In the current study, cognitive gamma responses showed higher amplitudes and prolonged latencies in AD patients compared to healthy controls. Overall, cognitive gamma latency responses were delayed over 100 ms in AD patients when compared to healthy controls. The latency gap of cognitive gamma responses between groups was most prominent in the 30–35 Hz frequency range. This delay in cognitive gamma activity may be related to lagged connections between limbic and association areas during memory and other cognition related processes, which are caused by neurodegeneration in AD. Additional explanation for these findings could be that there is a relationship between memory and gamma oscillatory responses. Another study reported that increased delta and gamma band responses along with increased gamma band connectivity in parieto-occipital regions was related to a memory task in healthy participants (Imperatori et al., 2014).

When the cognitive gamma responses were analyzed in the 0–800 ms time block, only an interaction effect was found, indicating higher responses in the 40–48 Hz frequency range in the right hemisphere in AD patients. However, when the time windows were divided and analyzed, a main group effect was found, showing higher amplitudes in AD patients. The earliest time window (0–200 ms) appeared to be important for cognitive gamma responses, as healthy controls exhibited the highest amplitude values over this particular time window, whereas AD patients continuously discharge gamma responses across all time windows. A possible explanation for higher amplitudes in AD could be related to this continuous gamma discharge. Analyzing multiple time windows made it possible to monitor gamma changes across time.

Previously reported data suggest that early components of the gamma response are more likely to relate to sensory functions and cognitive functions (Sakowitz et al., 2001, Senkowski et al., 2008). One study showed that phase-locked gamma activity was a part of the human auditory visual and auditory function (Başar et al., 1987), while another reported that a 40 Hz response in the first 100 ms had a sensory origin independent of cognitive tasks (Karakaş and Başar, 1998). In addition, an early response in the first 150 ms following stimulation was observed in the cortex, thalamus, hippocampus, and reticular formation of a cat brain (Demiralp et al., 1996). It has been shown that interwoven oscillatory activity, such as increased gamma and theta activity, allows for differential maintenance of temporal or spatial information during working memory tasks (Roberts et al., 2013). In another study, frontal theta phase and posterior gamma power demonstrated enhanced cross-frequency coupling during the encoding of visual stimuli; participants later remembered these stimuli while they forgot others (Friese et al., 2012). Moreover, emotional pictures or cognitive reappraisal tasks induce greater parietal gamma activity, suggesting that parietal gamma activity may be involved in the process of multiple cognitive reappraisals (Kang et al., 2012). Both experimental (Sakowitz et al., 2001) and human data (Senkowski et al., 2008) imply that there is an intersensory facilitation of gamma responses. Subcortical structures (e.g., superior colliculus) and higher cognitive areas (Başar et al., 2015) may be involved in the processes of multimodal convergence in the early time domain.

According to Fries (2009), it appears as though many different gamma band synchronization phenomena subserve many different functions. Fries also argues that gamma band synchronization is a fundamental process that subserves an elementary operation of cortical computation, which is in accordance with the findings of Basar (1980, 1998, 1999). However, Başar (2006) indicated that gamma band synchronization should be measured in many subcortical areas because there are many cognitive processing strategies during whole-brain operation. Therefore, gamma responses should be analyzed in a more detailed fashion, taking into consideration multiple time windows and frequency ranges.

### 4.3 Neuroanatomic basis of gamma responses

Target stimulation in the oddball paradigm elicits a compound response, including a sensory response and a cognitive response, in relation to a working memory task. The stimulating target induces an “attend” order. A light signal, including a cognitive load, can be tracked by the transmission of an electrical signal from the retina over the thalamic system to the occipital cortex. Cognitive responses use not only the simple visual pathway; the target signal is processed by bottom-up connections. It is expected that cognitive signal processing requires more time and occurs with a delay.

Earlier experimental studies reported that gamma oscillations exist in different parts of the cat brain (Başar-Eroglu and Basar, 1991, Başar, 1998, Başar, 1999, Başar, 2011). Several studies have estimated a post-stimulus hippocampal-cortical loop time of gamma activity in the range of 120–300 ms in experimental (Miller, 1991) and human studies (Dastjerdi et al., 2011). It is possible that there are several reverberations between association areas and the limbic system. These reverberations may also cause considerable delays in cognitive gamma responses in AD patients, which we believe may be due to neurodegenerative processes.

Every sensation in the brain induces cognitive activity, and all of the presented cognitive stimuli also evoke sensations. Globally-related connections can be summarized as 1) “Purely sensory connections” to the cortex over the thalamic nuclei; 2) “Secondary connections” to the cortex over the reticular formation; 3) “Secondary connections” over the limbic system; and 4) “Connections within the cortex” between association areas (Başar et al., 2015).

The present study once more indicates that multiple gamma windows exist in different time and frequency domains (Başar, 2013, Başar et al., 2015). However, the functionality of these different gamma windows remains unclear.

### 4.4 Concluding remarks

In this paper, we report that the latency of cognitive gamma responses was delayed about 100 ms in AD patients. This delay was most likely due to delays in propagation, reverberation of signals, or recurrent excitation. Since it has been reported that all signals are conveyed to the hippocampal and heteromodal cortical areas, brain degeneration and related atrophy may cause the delay in the transmission of signals in AD/MCI patients (Yener et al., 2015).

Furthermore, we described globally separated sensory and cognitive gamma responses between AD patients and healthy controls. In the future, we may be able to make more precise statements regarding specific functions, as these experiments can be performed by modifying the function related to the stimulus. It is almost imperative to use a pure sensory stimulation (with the same luminance or sound level) as a baseline to separate sensory components from more complex cognitive responses (Başar et al., 2015).

An approach that takes into consideration time and frequency windows is important for gamma frequency ranges. For gamma band analyses, a different approach was needed to investigate the differences between groups. A matrix of time domains and frequency windows may help to understand the underlying time-based differences of brain dynamics in AD patients and healthy controls.

Finally, gamma activity in multiple frequency bands over several time windows may add additional value to our and other researchers' previous findings. The present report aims to add an additional electrophysiological biomarker to the Alzheimer's literature. Altogether, multiple electrophysiological biomarkers specific to AD can be listed:

1) Decrease in delta responses, especially in a cognitive paradigm for both visual and auditory stimulations (Caravaglios et al., 2008, Polikar et al., 2007, Yener et al., 2008, Yener et al., 2012, Yener et al., 2013, Kurt et al., 2014) and its relation to frontal atrophy as an index of neurodegeneration (Yener et al., 2015).
2) Decrease of event-related delta and theta coherence values, especially in a cognitive paradigm, which showed significant connectivity deficits in AD (Güntekin et al., 2008, Basar et al., 2010).
3) Decrease of theta event-related phase-locking in AD (Yener et al., 2007).
4) In addition to the listed results, the present study showed that AD patients had reduced early sensory gamma responses and delayed cognitive gamma responses. Overall, cognitive gamma latency responses were delayed over 100 ms in AD patients when compared to healthy controls.

## Figures and Tables

**Table 1:** General demographic and clinical features of participants.

	Healthy controls (N = 15)	AD patients (N = 15)	p
Age (SD)	67.47 (4.14)	67.53 (6.48)	0.973^a^
Education (SD)	8.73 (6.03)	8.67 (4.75)	0.973^a^
Gender (M/F)	8/7	8/7	1.000^b^
MMSE (SD)	28.73 (2.02)	21.85 (3.46)	0.000^a^

SD: standard deviation, M: male, F: female, AD: Alzheimer's disease, MMSE: mini-mental state examination

a Independent sample t-test.

b Chi-square.

**Table 2:** ANOVA results of the maximum peak-to-peak amplitude and latency of event-related and evoked gamma oscillations.

Effects	F	df effect	df error	p	Greenhouse–Geisser adjusted p-value	Greenhouse–Geisser epsilon
*Maximum peak-to-peak gamma SEO amplitudes at three frequency ranges over a 0–800 ms time window*						
FR	14.112	2	56	0.000	0.000	0.924

*Maximum peak-to-peak gamma SEO amplitudes at three frequency ranges over four time windows*						
FR	17.244	2	56	0.000	0.000	0.589
TW	10.454	3	84	0.000	0.000	0.803
AP	14.203	1	28	0.001	0.001	1.000
FR × TW	2.956	6	168	0.009	0.037	0.505
FR × AP	13.685	2	56	0.000	0.000	0.588
FR × TW × LAT × GROUP	2.503	12	336	0.004	0.03	

*Maximum peak-to-peak gamma target ERO amplitudes at three frequency ranges over a 0–800 ms time window*						
FR	14.569	2	56	0.000	0.000	0.612
FR × LAT × GROUP	3.379	4	112	0.012	0.022	

*Maximum peak-to-peak gamma target ERO amplitudes at three frequency ranges over four time windows*						
GROUP	4.259	1	28		0.048	
FR	8.790	2	56	0.000	0.004	0.589
TW	11.146	3	84	0.000	0.000	0.750
TW × GROUP	4.744	3	84	0.004	0.009	
FR × TW	6.921	6	168	0.000	0.000	0.500

*Gamma SEO latency at three frequency ranges over a 0–800 ms time window*						
AP × LAT	3.411	2	56	0.04	0.042	0.960

*Gamma target ERO latency at three frequency ranges over a 0–800 ms time window*						
GROUP	6.132	1	28		0.02	
FR	3.645	2	56	0.033	0.038	0.898

FR: frequency range, TW: time window, AP: anterior–posterior, LAT: laterality.
